# Institutionalization of Health Technology Assessment of medical devices: a cluster analysis of EU, EEA, and EFTA countries

**DOI:** 10.1017/S0266462325100251

**Published:** 2025-07-08

**Authors:** Mario Cesare Nurchis, Gian Marco Raspolini, Pietro Derrico, Carlo Favaretti, Matteo Ritrovato, Giandomenico Nollo, Gianfranco Damiani

**Affiliations:** 1 Department of Life Science, Health, and Health Professions, Università degli Studi Link, Rome, Italy; 2 Department of Health Science and Public Health, Section of Hygiene, Università Cattolica del Sacro Cuore, Rome, Italy; 3 Società Italiana di Health Technology Assessment (SIHTA), Rome, Italy; 4 ConsulHTA, Rome, Italy; 5 Leadership in Medicine Research Center, Università Cattolica del Sacro Cuore, Rome, Italy; 6 Bambino Gesù Children’s Hospital, IRCCS, Rome, Italy; 7 Department of Industrial Engineering, University of Trento, Trento, Italy; 8 Department of Woman and Child Health and Public Health, Fondazione Policlinico Universitario Agostino Gemelli IRCCS, Rome, Italy

**Keywords:** technology assessment, biomedical, equipment and supplies, Europe, policy making, cluster analysis

## Abstract

**Objectives:**

Health technology assessment of medical devices (HTA-MDs) presents unique challenges compared to pharmaceuticals. Total MD expenditure continues to grow in Europe, and countries typically conduct their own HTA-MDs evaluations, with varying institutionalization arrangements. European Union’s (EU’s) HTA Regulation aims to establish collaborative clinical assessments across Member States, potentially expediting the path from EU safety certification of MDs to pricing and reimbursement decisions. This study aims to identify emergent configurations among institutionalizations of HTA-MDs in the EU, European Economic Area (EEA), and European Free Trade Association (EFTA) countries.

**Methods:**

Publicly available data were cross-sectionally collected for EU, EEA, and EFTA countries until August 2024 to allow a cross-country analysis of HTA-MDs institutionalizations. Countries were included if they had at least one publicly mandated body for HTA-MDs. Data sources were scientific databases, institutional websites, and HTA bodies’ documentation. A framework of 16 elements, qualitatively describing the institutionalization of HTA-MDs, was developed based on a document review and used as a dataset for agglomerative hierarchical cluster analysis to identify patterns of HTA-MDs institutionalization.

**Results:**

The 21 included countries formed three clusters: Cluster 1 featured regulatory-focused, legally bound HTA-MDs systems with mandatory assessments determining reimbursement decisions; Cluster 2 was characterized by regulatory functions, external expert collaboration, formal prioritization processes, and organized Horizon Scanning; Cluster 3 showed recommendatory functions, nonmandatory assessments, and limited impact on reimbursement decisions.

**Conclusions:**

HTA-MDs institutionalizations could benefit from implementing prioritization processes of evaluations, establishing networks of collaborative assessment centers, and ensuring links between evaluations and reimbursement decisions.

## Background

Medical devices (MDs) are products or equipment intended for a medical purpose, representing a broad category of health technologies. When compared to pharmaceuticals, MDs have features that make the process of assessing their value for health through health technology assessment (HTA) more challenging, these generally being the lower availability of evidence, user-dependency of performance, shorter product life cycle, and less explicit target populations and clinical outcomes (1).

Despite the relative difficulties for HTAs, expenditure for MDs from public and private buyers has been growing in Europe (2), which can only increase the interest of decision makers with resource constraints in having evidence on their side when investing or disinvesting public money on MDs and on procedures, systems, and programs involving these. To address such demands for information, decision makers should be able to rely on adequate human and organizational capacity for systematic HTA of MDs (HTA-MDs) production (3).

The Regulation on HTA (HTAR) of the European Union (EU) (4) aims to decrease the workload of individual HTA bodies, establishing a system for the joint assessment of clinical aspects of selected “high-risk” MDs, being those belonging to Class IIb or III, and to Class D in the case of in vitro diagnostic MDs. Evaluations performed under the HTAR will address only clinical aspects of technologies, such as relative effectiveness and safety, thereby not constituting value appraisals. Clinical aspects were privileged for their greater generalizability, compared to nonclinical aspects. The desired outcome of this infrastructural collaborative system is to shorten the average time interval between the approval of a technology at the EU level through CE marking and its potential entry into Member States’ pricing and reimbursement frameworks.

Currently, the tendency among EU countries is to individually produce HTA-MDs reports, whose utilization by decision makers in the respective healthcare systems reflects the characteristics of institutionalization of HTA-MDs. Defining the latter as “the process of conducting and utilizing HTA[-MDs] as a normative practice for guiding healthcare priority-setting processes”, (5) it can be described for each country where HTA-MDs are performed by a set of common elements, that is, variables, and the different modalities they can assume.

Taxonomies and interpretative schemes delineating the elements of institutionalization of HTA were exhibited in documents released by international organizations, such as the World Health Organization (WHO) (6), Health Technology Assessment International (HTAi), and ISPOR – The Professional Society for Health Economics and Outcomes Research (7), and published in the scientific literature (8;9).

The objective of this study was to identify emergent configurations within the institutionalizations of HTA-MDs in the EU, European Economic Area (EEA), and European Free Trade Association (EFTA) countries.

## Methods

The study planned to use only aggregated data, fully complying with the Helsinki Declaration of Ethical Principles and adhering to Italian (Law 2003/196) and international (EC/2016/679) data protection regulations.

### Study design and target population

This is a cross-sectional study based on data publicly available on institutional datasets and scientific literature. Data were searched for the included countries until 31 August 2024.

The target population consisted of the countries belonging to the EU, EEA, and EFTA. The rationale behind the selection of such countries is motivated by the high degree of institutional integration through common legal and regulatory frameworks and by aligned economic policies.

### Framework model

A report by the WHO (6) and a peer-reviewed guidance by HTAi and ISPOR (7) were exploited as methodological, taxonomic, and interpretative sources. The logical framework for HTA mechanisms by the WHO was chosen as the foundational structure for a descriptive model of HTA-MDs systems and governance arrangements. Specifically, the elements pertaining to the “Inputs” and “Activities” blocks of the WHO framework were integrally retained. Then, specific elements from relevant scientific articles were extracted (8;9). Particularly, from Fontrier et al.’s “Conceptual framework outlining type, scope, and nature of HTA activities,” the elements HTA governance, type of organization, HTA role, HTA scope and geographical coverage, HTA model, assessment versus appraisal, stakeholder involvement, HTA recommendations, and funding decisions were retrieved. From Tarricone et al., the following elements were collected: prioritization process, assessment process, appraisal process, final decision and appeal, and the impact of HTA recommendations.

The final integrated framework model consisted of 16 elements descriptive of HTA-MDs institutionalization. To reflect possible arrangements, several modalities were formulated for each element. The elements and their modalities are presented in Table 1. Their meaning is further outlined in Supplementary Materials.

**Table 1.** Framework of the institutionalization of health technology assessment of medical devices in a country

No.	Element of HTA-MD institutionalization	Element modality				Source
i	Impact of HTA-MDs recommendations with regard to funding decisions	Binding results of HTA-MDs	Nonbinding results of HTA-MDs			(6;8;9)
ii	Basis for national HTA-MD institutionalization	Technical procedural documents	Legal acts formalizing procedural documents			(6)
iii	The body entrusted with the public HTA-MD mandate can commission external experts for assessment and/or appraisal activities	The body can commission external experts for assessments and/or appraisals, and it can run these activities in-house as well	The body cannot commission external experts; rather, it relies solely on in-house production of assessments and/or appraisals			(6;9)
iv	Nature of the body entrusted with the national mandate for HTA-MD activities	Governmental	Academic	Healthcare provider	Independent	(6;8)
v	Type of healthcare system	Beveridge	Bismarck	National Health Insurance		(6)
vi	Prevailing level of HTA-MD activities	National	Subnational (region, province, and county)	Local		(8)
vii	Funds allocated specifically for HTA-MD system implementation (technical/organizational structures and/or HTA-MDs activities)	The system is financed through funds specifically allocated for HTA-MDs	No funds are specifically allocated for the HTA-MD system			(6)
viii	HTAs are mandatory before the decision whether MDs, or the procedures/systems/programs in which they are employed, are eligible for reimbursement/funding	An HTA evaluation is necessary for determining reimbursability or allocating funds	Even without an available HTA evaluation, reimbursability can be determined, and funds can be allocated			(6)
ix	The HTA-MD body is responsible for the decision on pricing and/or reimbursement tariffs	Pricing	Reimbursement	Pricing and reimbursement	None	(6)
x	Consultations with external stakeholders to guide the design of methodologies	Stakeholders (e.g., clinicians, healthcare experts, patients, and developers) external to the HTA-MD body are consulted to guide the design of the methodologies of evaluations	Methodologies are designed without the consultation of stakeholders external to the HTA-MD body			(6;8)
xi	Kinds of domains of MDs analyzed	Clinical aspects	Nonclinical aspects	Clinical and nonclinical aspects		(6;8;9)
xii	Publicly available assessment and/or appraisal reports	Only assessment reports become publicly available	Only appraisal reports become publicly available	Both assessment and appraisal reports become publicly available	Assessments and/or appraisal reports do not become publicly available	(6;9)
xiii	Education activities to HTA-MD stakeholders provided by the national HTA body	The HTA-MD body organizes educational activities on HTA-MDs aimed at stakeholders involved in HTA	The HTA-MD body does not organize educational activities on HTA-MDs aimed at stakeholders involved in HTA			(6)
xiv	Conduction of a systematic process of priority setting of HTA-MD evaluations (existence of an ordered list; systematic topic identification, selection, and prioritization)	HTA-MD evaluations are initiated following an order resulting from processes of topic identification, selection, and prioritization	The order of initiation of HTA-MD evaluations is not systematically determined			(6;9)
xv	Conducting a systematic process of horizon scanning for MDs	Horizon scanning activities for MDs are systematically conducted	Horizon scanning activities for MDs are not systematically conducted			(6;9)
xvi	Designated appeal mechanisms are in place against HTA-MD outcomes (placed during/after the assessment process and/or the deliberative process)	Designated mechanisms are in place to allow stakeholders to appeal the contents of HTA-MD reports or decisions concerning evaluated technologies	There are no designated mechanisms through which stakeholders may formally appeal the contents of HTA-MD reports or decisions concerning evaluated technologies			(6;9)

HTA-MDs, health technology assessment of medical devices.

### Search strategy and data sources

In order to assign the correct modality for each framework element for the investigated countries, scientific databases (i.e., MEDLINE, Web of Science, and Scopus) were initially queried to obtain peer-reviewed data on the institutionalization of HTA-MDs in the EU, EEA, and EFTA countries.

To retrieve more data, institutional websites, including those of supranational and national bodies (e.g., WHO, EU commission, European Observatory on Health Systems and Policies, health ministries, and social health insurance institutions), were referred to.

Afterward, the websites of international networks and societies involved in HTA and related fields (e.g., HTAi, ISPOR, and the former European Network for Health Technology Assessment (EUnetHTA)) were examined for additional information.

The official websites of HTA agencies, units, or committees in the selected countries were accessed to gather insights about the latest HTA-MDs systems and governance arrangements to validate the retrieved information and to incorporate any missing information.

For each country, data collection continued until all modalities had been assigned and the screening of official HTA agency, unit, or committee websites yielded no updates beyond the data already collected. Information was sourced in both English and the official language of each country. Search terms used are provided in the Supplementary Materials.

Countries were included if, within them, at least one HTA body was entrusted with a mandate from a public institution (e.g., central or federal government, ministry, regional government, public insurance body, and public healthcare provider) to produce assessments and/or appraisals of any kind of MD, either directly or via third-party commissioning.

### Dataset construction

The framework in Table 1 was used as the structure for a dataset used in subsequent analysis. The dataset was populated with the modalities of the elements for each country that met the inclusion criterion. Included and excluded countries were reviewed by two authors independently to validate the appropriateness of these selections.

### Statistical analysis

In machine learning, algorithms are generally classified into two main types: (a) supervised and (b) unsupervised. The former focuses on learning the relationship between input data and the target variable, while the latter is designed to uncover hidden patterns within a dataset. Cluster analysis, a key method within unsupervised learning, seeks to group *n* observations into *K* clusters, where observations within the same cluster are more similar to each other than to those in different clusters. Among the popular clustering techniques are K-Means and hierarchical clustering (HCA). For the present analysis, agglomerative HCA was applied to group countries that showed the greatest similarity across the studied modalities. Compared to qualitative analyses, clustering enables grouping based on multiple characteristics simultaneously. HCA reveals latent structures that might not be evident through thematic analysis. Agglomerative HCA was chosen for its widespread use and intuitive approach over divisive methods. Given the mixed nature of the dataset, we employed the Gower distance metric, which is well-suited for handling both categorical and continuous variables. Clustering was performed using the complete linkage method, which merges clusters based on the maximum pairwise distance between observations in different clusters.

To determine the optimal number of clusters, the silhouette score and the elbow method were employed. The silhouette score evaluates how similar an observation is to its own cluster compared to other clusters, with values closer to 1 indicating better-defined clusters. Iterating over a range of potential cluster counts, the silhouette score was calculated for each configuration to identify the one maximizing such score. Furthermore, the elbow method, which examines the within-cluster sum of squares (WSS) as a function of the number of clusters, was adopted. By plotting the WSS against the number of clusters, the elbow point, where the rate of reduction in WSS diminishes, is identified. Countries were categorized into *K* = 3 clusters. This cutoff height was selected to balance minimizing within-cluster variance while avoiding overclustering, ensuring that the resulting clusters were interpretable and meaningful in the context of the analysis.

The dendrogram was plotted to provide a visual representation of how countries were merged at each step of the clustering process. Dendrograms visually depict how observations are grouped at different levels of (dis)similarity. At the lowest level, each observation is considered as its own cluster. Vertical lines represent these observations, which gradually merge as their (dis)similarity decreases. A horizontal line connects two observations when they are grouped together, and this process continues until all observations are combined into a single cluster at the top of the diagram. The height of the vertical lines and the scale of the (dis)similarity axis provide insight into the clustering strength. Longer vertical lines indicate greater separation between clusters, especially at higher levels of the dendrogram, suggesting well-defined groupings. In contrast, shorter vertical lines imply that the clusters are not as distinct. To provide a comprehensive assessment of the clustering quality, the C-index was adopted. This index evaluates the intracluster dispersion (how spread out the points within a cluster are) and the intercluster separation (how far apart clusters are from each other). According to the C-index, values closer to 0 indicate tight clusters while values near 1 indicate poor clustering.

The characteristics of the groups of countries obtained were described narratively and in table form, reporting the most recurring modalities within each group for each element of institutionalization, reflecting the order in which the elements are listed in Table 1. Moreover, for each group, the modalities shared by all the countries within the group were reported.

The statistical software R (version 4.2.2) was used for all statistical analyses.

## Results

The analysis included 21 countries meeting the inclusion criteria, which are: Austria, Belgium, Croatia, Denmark, Estonia, Finland, France, Germany, Hungary, Ireland, Italy, Latvia, Lithuania, Norway, Poland, Portugal, Slovakia, Spain, Sweden, the Swiss Confederation, and the Netherlands.

However, Bulgaria, Cyprus, Czech Republic, Greece, Iceland, Liechtenstein, Luxembourg, Malta, Romania, and Slovenia were excluded since, at the time of our research, their public HTA agencies, including any national or regional/local coordination bodies, do not conduct a documented formal evaluation process of MDs.

**Figure 1.**
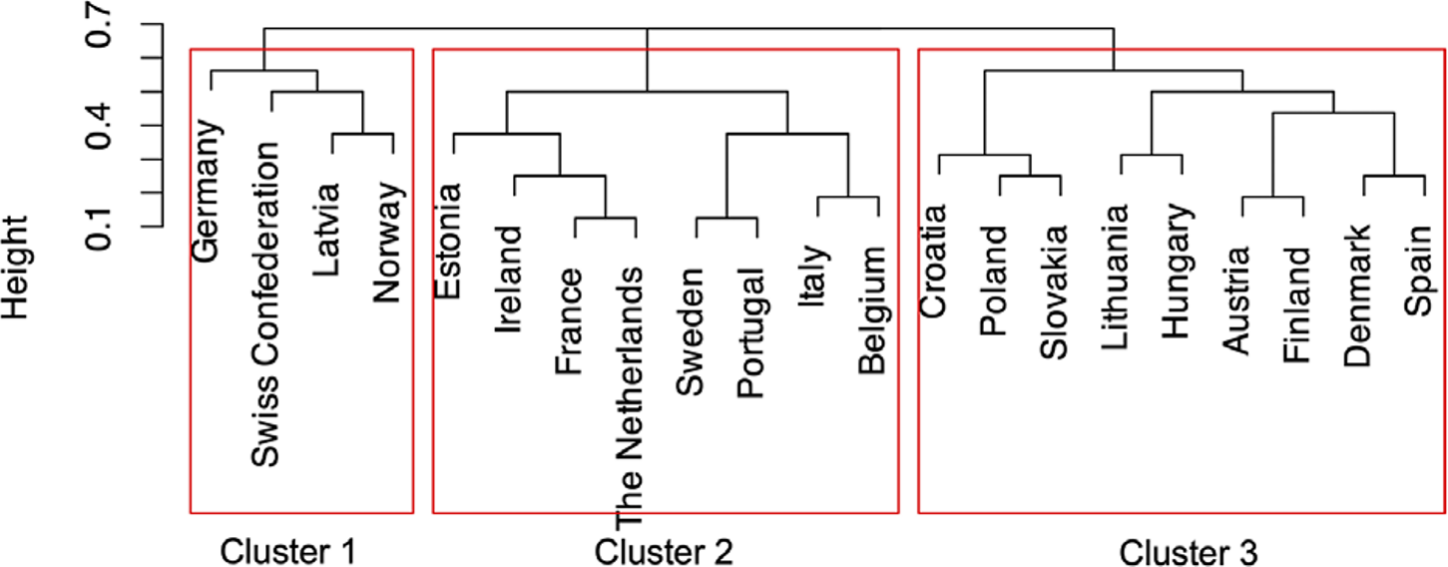
Cluster dendrogram of European Union, European Economic Area, and European Free Trade Association countries according to their modalities of institutionalization of health technology assessment of medical devices. The *y*-axis (height) represents the distance or dissimilarity measure at which clusters are merged. Greater height values indicate greater dissimilarity between connected groups. Clusters that merge at lower positions are more similar to each other, while those joining at higher positions represent more distinct groupings in the dataset.

Figure 1 depicts the dendrogram representing the three groups or clusters that were identified.

Cluster 1 includes Germany, Latvia, Norway, and the Swiss Confederation. These countries predominantly manifest the following characteristics: HTA-MDs with a (i) regulatory function, (ii) bounded by specific legal acts, conducted (iii) within the HTA-MDs (iv) governmental body, in a healthcare system based on (v) social health insurance or a National Health Service. HTA-MDs evaluations are (vi) nationally centralized and (vii) funded through allocations for HTA-MDs. Also, they are (viii) mandatory and determinant for (ix) reimbursement eligibility. The methodologies are developed (x) with contributions from stakeholders and address (xi) both clinical and nonclinical aspects, with (xii) assessment reports publicly available. (xiii) The national HTA body (xiii) does not provide any HTA-MDs courses. There is (xiv) no formal prioritization of HTA-MDs evaluations (xv) or organized Horizon Scanning activities. (xvi) Designated mechanisms are in place for appealing HTA-MDs outcomes.

The only modality shared by all the countries of Cluster 1 was the (i) regulatory function of HTA-MDs.

Cluster 2 comprises Belgium, Estonia, France, Ireland, Italy, Portugal, Sweden, and the Netherlands. It shows countries characterized by the following aspects: an HTA-MDs with a (i) regulatory function, (ii) bounded by specific legal acts, conducted (iii) by collaborative centers commissioned by the HTA-MDs (iv) governmental body, within a healthcare system based on (v) social health insurance or a National Health Service. HTA-MDs evaluations are (vi) nationally centralized and (vii) funded through allocations for HTA-MDs. Furthermore, they are (viii) mandatory and determinant for (ix) reimbursement eligibility and prices. The methodologies are developed (x) with contributions from several stakeholders and address (xi) both clinical and nonclinical aspects, with (xii) assessment and appraisal reports publicly available. The national HTA body (xiii) offers courses on HTA-MDs. There is (xiv) formal prioritization of HTA-MDs evaluations and (xv) organized Horizon Scanning procedures. (xvi) Designated mechanisms are in place for appealing HTA-MDs outcomes.

Modalities common to all countries of Cluster 2 were the (xii) public availability of assessment and appraisal reports, the HTA-MDs activities performed prevalently at a (vi) national level, the (iii) commissioning of external experts for assessment and/or appraisal activities by the body entrusted with the public HTA-MDs mandate, the presence of (vii) funds allocated specifically for HTA-MDs system implementation, and the (x) consultation of external stakeholders (e.g., clinicians, healthcare experts, patients, and developers) to inform the design of methodologies.

Cluster 3 includes Austria, Croatia, Denmark, Finland, Hungary, Lithuania, Poland, Slovakia, and Spain. Such countries mainly exhibit the following features: an HTA-MD with a (i) recommendatory function, (ii) not bounded by specific legal acts, conducted (iii) within the HTA-MD (iv) governmental body of a healthcare system based on (v) social health insurance. HTA-MDs evaluations are (vi) nationally centralized and (vii) funded through allocations for HTA-MDs, but (viii) not mandatory and (ix) not determinant for reimbursement or pricing decisions. The methodologies are developed (x) with contributions from various stakeholders and address (xi) both clinical and nonclinical aspects, with (xii) assessment and appraisal reports publicly available. (xiii) Courses on HTA-MDs are provided by the national HTA agency. There is (xiv) no formal prioritization of HTA-MDs evaluations (xv) or organized Horizon Scanning activities. (xvi) There are no designated mechanisms for appealing the outcomes of HTA-MD reports.

Only one modality was shared by all the countries in Cluster 3, namely the (ix) HTA-MD bodies not being responsible for decisions on pricing and/or reimbursement tariffs.

For illustrative purposes, HTA-MDs institutionalizations of three countries, described using the framework developed for this study, are reported in the Supplementary Materials.

Table 2 provides a summary of the most recurring modalities within each Cluster.

**Table 2.** Most recurring modalities within each group for each element of institutionalization

No.	Element of HTA-MD institutionalization	Most recurring modality for Cluster 1	Most recurring modality for Cluster 2	Most recurring modality for Cluster 3
i	Impact of HTA-MDs recommendations with regard to funding decisions	Binding results of HTA-MDs	Binding results of HTA-MDs	Nonbinding results of HTA-MDs
ii	Basis for national HTA-MD institutionalization	Legal acts formalizing procedural documents	Legal acts formalizing procedural documents	Technical procedural documents
iii	The body entrusted with the public HTA-MD mandate can commission external experts for assessment and/or appraisal activities	The body cannot commission external experts; rather, it relies solely on in-house production of assessments and/or appraisals	The body can commission external experts for assessments and/or appraisals, and it can run these activities in-house as well	The body cannot commission external experts; rather, it relies solely on in-house production of assessments and/or appraisals
iv	Nature of the body entrusted with the national mandate for HTA-MD activities	Governmental	Governmental	Governmental
v	Type of healthcare system	Beveridge or Bismarck	Beveridge or Bismarck	Bismarck
vi	Prevailing level of HTA-MD activities	National	National	National
vii	Funds allocated specifically for HTA-MDs system implementation (technical/organizational structures and/or HTA-MDs activities)	The system is financed through funds specifically allocated for HTA-MDs	The system is financed through funds specifically allocated for HTA-MDs	The system is financed through funds specifically allocated for HTA-MDs
viii	HTAs are mandatory before the decision whether MDs, or the procedures/systems/programs in which they are employed, are eligible for reimbursement/funding	An HTA evaluation is necessary for determining reimbursability or allocating funds	An HTA evaluation is necessary for determining reimbursability or allocating funds	Even without an available HTA evaluation, reimbursability can be determined, and funds can be allocated
ix	The HTA-MD body is responsible for the decision on pricing and/or reimbursement tariffs	Reimbursement	Pricing and reimbursement	None
x	Consultations with external stakeholders to guide the design of methodologies	Stakeholders (e.g., clinicians, healthcare experts, patients, and developers) external to the HTA-MD body are consulted to guide the design of the methodologies of evaluations	Stakeholders (e.g., clinicians, healthcare experts, patients, and developers) external to the HTA-MD body are consulted to guide the design of the methodologies of evaluations	Stakeholders (e.g., clinicians, healthcare experts, patients, and developers) external to the HTA-MD body are consulted to guide the design of the methodologies of evaluations
xi	Kinds of domains of MDs analyzed	Clinical and nonclinical aspects	Clinical and nonclinical aspects	Clinical and nonclinical aspects
xii	Publicly available assessment and/or appraisal reports	Only assessment reports become publicly available	Both assessment and appraisal reports become publicly available	Both assessment and appraisal reports become publicly available
xiii	Education activities to HTA-MD stakeholders provided by the national HTA body	The HTA-MD body does not organize educational activities on HTA-MDs aimed at stakeholders involved in HTA	The HTA-MD body organizes educational activities on HTA-MDs aimed at stakeholders involved in HTA	The HTA-MD body organizes educational activities on HTA-MDs aimed at stakeholders involved in HTA
xiv	Conduction of a systematic process of priority setting of HTA-MD evaluations (existence of an ordered list; systematic topic identification, selection, and prioritization)	The order of initiation of HTA-MDs evaluations is not systematically determined	HTA-MD evaluations are initiated following an order resulting from processes of topic identification, selection, and prioritization	The order of initiation of HTA-MD evaluations is not systematically determined
xv	Conducting a systematic process of horizon scanning for MDs	Horizon scanning activities for MDs are not systematically conducted	Horizon scanning activities for MDs are systematically conducted	Horizon scanning activities for MDs are not systematically conducted
xvi	Designated appeal mechanisms are in place against HTA-MD outcomes (placed during/after the assessment process and/or the deliberative process)	Designated mechanisms are in place to allow stakeholders to appeal the contents of HTA-MD reports or decisions concerning evaluated technologies	Designated mechanisms are in place to allow stakeholders to appeal the contents of HTA-MD reports or decisions concerning evaluated technologies	There are no designated mechanisms through which stakeholders may formally appeal the contents of HTA-MD reports or decisions concerning evaluated technologies

*Note*: Cluster 1 includes Germany, Latvia, Norway, and the Swiss Confederation. Cluster 2 includes Belgium, Estonia, France, Ireland, Italy, Portugal, Sweden, and the Netherlands. Cluster 3 includes Austria, Croatia, Denmark, Finland, Hungary, Lithuania, Poland, Slovakia, and Spain.

HTA-MDs, health technology assessment of medical devices.

Relative to the model fit, the C-index amounted to 0.24, suggesting moderately good clustering.

## Discussion

The HCA identified three clusters. Among these, Cluster 2 emerged as a qualified profile to steer HTA-MD policy making toward more prepared institutionalization models. Cluster 2’s profile stands out due to factors indicating a firmer institutional commitment to promote the role of HTA-MDs in decision making, such as the formal prioritization of evaluations, as well as the direct influence on policies and practices at different levels because of the regulatory role of HTA-MDs and the mandate for reimbursement – and pricing-aimed evaluations.

This analysis attempted to identify patterns in the combinations of HTA policies among countries.

Other studies, as well, have addressed research questions focused on elements of HTA institutionalization internationally. They are discussed comparatively hereafter, and recommendations are provided.

Fontrier et al. (8) investigated the characteristics of HTA systems in Europe and hypothesized the ways they affect the funding of technologies. Some of our findings confirmed the national prevailing level of overall HTA activities and the attention to address both clinical and nonclinical aspects. Indeed, a decentralized production of HTA reports would not only lead to a multiplication of evaluation efforts for the same technologies but would also require an unrealistic increase in the number of employed assessors, the latter requiring various sought-after competencies. For these reasons, a centralization of HTA evaluation efforts is suggested to promote the long-term sustainability of the system.

Ormstad et al. (10) mapped Horizon Scanning systems for MDs, also known as Early Awareness and Alert Systems. Their results confirm that such organized systems are not widespread in Europe. This may be because existing international collaborations in this field reduce the utility of country-level Horizon Scanning systems. Participation in joint Horizon Scanning activities, like those mandated in the HTAR (4), is, however, advised for improving the preparedness of stakeholders for a more efficient and effective financial and organizational planning, health service research prioritization, and controlled diffusion of MDs.

A report by the European Commission analyzing the HTA-MDs of the EU, the United Kingdom, and Norway (11) reported that roughly half of the countries had evaluations mandatorily performed for MDs’ reimbursement-funding decisions. Similarly, our study found that 10 out of the 21 countries included shared this practice.

Decision makers, to enhance their ability to draw public trust, should rely on mandatorily produced HTA documents, thus ensuring value-based investments and disinvestments, as well as fostering an equitable distribution of health benefits across the population.

The European Commission report (11) also identified that three-quarters of HTA-MDs bodies in the sample turned to commissioning external experts for assessment and/or appraisal activities. Similarly, in our investigation, the majority (~60 percent) of included countries can resort to collaborative centers. National networks of centrally accredited collaborative centers, overseen by national bodies with the mandate for HTA-MDs, could support health systems with a high demand for evaluations of MDs and constraints in centralized resources. A hypothetical industry self-assessment mechanism, modeled after notified bodies (12) (see Article 120, paragraph 3, letter a of Regulation (EU) 2017/745 and Article 113, paragraph 3, letter b of Regulation (EU) 2017/746), could allow the developer to select the assessor, with the assessor undergoing periodic audits and facing repercussions, along with the developer, in the event of a negative audit outcome. This approach could foster a competitive environment, leading developers to prefer assessors with high-quality standards, improving both the quality and quantity of HTA-MDs in the country.

Furthermore, an analysis of former EUnetHTA partner countries found that approximately half of them had HTA-MDs agencies with formal criteria in place for the identification and selection of MDs and prioritization of evaluations. There is no evidence that priority-setting processes could be beneficial for every HTA system or context (13). Nevertheless, a centralized formal priority setting could favor transparency in gauging MD candidates for reimbursement, in contrast to systems skewed in favor of MDs from developers with greater capacity to independently commission and deliver HTA reports.

With the advent of the HTAR, it is reasonable to expect that EU countries will try to evolve toward HTA-MDs configurations more sustainable and effective for performing nonclinical evaluations complementary to the “clinical” reports centrally produced at the European level. This evolution cannot take place without a serious effort at the national level in educating capable assessors, allocating coherent funds for HTA-MDs implementation and activities, and outlining the governance of production. The major limitation of this study is the relatively small sample size and the nature of the elements (i.e., variables), which may undermine the performance of the HCA. However, assumptions of the analysis and the model’s goodness of fit were thoroughly checked. In addition, the reduced sample size may be justified by the strict need to exclude (a) countries not performing HTA-MDs and (b) countries likely not participating in the forthcoming HTAR-based system. Another caveat is that possible diversified HTA-MDs policies within the same country for different types of MDs were not taken into account due to the paucity of publicly available national-level information.

Additional research is needed to understand how the diverse profiles of HTA-MDs’ institutionalizations impact sustainable health. Further work could replicate the methodology for other regions of the world and even for the HTA of pharmaceuticals or other health technologies.

## Conclusions

The HCA of the modalities of institutionalization of HTA-MDs in the EU, EEA, and EFTA countries identified three clusters, corresponding to three different institutionalization profiles. Each profile is characterized by a different governance model of HTA-MDs evaluations, with Cluster 2 (i.e., Belgium, Estonia, France, Ireland, Italy, Portugal, Sweden, and the Netherlands) standing out for factors suggesting firmer institutional commitment to HTA-MDs processes, reflecting the political validation granted to HTA by the EU HTAR. The analysis utilized a taxonomical framework to describe national HTA-MDs institutionalizations, which enabled the characterization of each included country.

## Supporting information

Nurchis et al. supplementary material Nurchis et al. supplementary material

## Supplementary material

The supplementary material for this article can be found at http://doi.org/10.1017/S0266462325100251.
