# Supplementary material for: Institutionalization of Health Technology Assessment of medical devices: a cluster analysis of EU, EEA, and EFTA countries
Source: Int J Technol Assess Health Care. 2025 Jul 8;41(1):e44. doi: 10.1017/S0266462325100251 (PMC12303692; doi:10.1017/S0266462325100251)
Supplement: Nurchis et al. supplementary material [file S0266462325100251sup001.docx]

**Supplementary materials**

**Contents of this document:**

1. **Search terms and queries used**
2. **Clarifications on the elements of the *framework of the institutionalization of Health Technology Assessment of medical devices in a country***
3. **Institutionalization of Health Technology Assessment of medical devices in a country for each Cluster**
4. **Search terms and queries used**

The search terms below were used. These search terms were interpolated through Boolean operators to build search queries for MEDLINE/PubMed, Web of Science, and Scopus.

**Search terms:**

*health technology assessment; HTA; value assessment; comparative assessment; appraisal; medical device; medical technology; medtech; equipment; procedure; policy; regulation; legislation; law; statute; institutionalization; governance; pricing; reimbursement; funding; financing; spending; horizon scanning; horizon scan; early awareness; alert system*

**Search queries for MEDLINE/PubMed, Web of Science, and Scopus:**

For **MEDLINE/PubMed**:

(“health technology assessment”[Title/Abstract] OR HTA[Title/Abstract] OR “value assessment”[Title/Abstract] OR “comparative assessment”[Title/Abstract] OR appraisal[Title/Abstract] OR appraisals[Title/Abstract])

AND

(“medical device”[Title/Abstract] OR “medical devices”[Title/Abstract] OR “medical technology”[Title/Abstract] OR “medical technologies”[Title/Abstract] OR medtech[Title/Abstract] OR equipment[Title/Abstract] OR procedure[Title/Abstract] OR procedures[Title/Abstract])

AND

(policy[Title/Abstract] OR policies[Title/Abstract] OR regulation[Title/Abstract] OR regulations[Title/Abstract] OR legislation[Title/Abstract] OR law[Title/Abstract] OR laws[Title/Abstract] OR statute[Title/Abstract] OR statutes[Title/Abstract] OR institutionalization[Title/Abstract] OR governance[Title/Abstract])

AND

(pricing[Title/Abstract] OR reimbursement[Title/Abstract] OR funding[Title/Abstract] OR financing[Title/Abstract] OR spending[Title/Abstract] OR "horizon scanning"[Title/Abstract] OR "horizon scan"[Title/Abstract] OR "early awareness"[Title/Abstract] OR "alert system"[Title/Abstract])

For **Web of Science**:

TS=(“health technology assessment” OR HTA OR “value assessment” OR “comparative assessment” OR appraisal OR appraisals)

AND

TS=(“medical device” OR “medical devices” OR “medical technology”” OR “medical technologies” OR medtech OR equipment OR procedure OR procedures)

AND

TS=(policy OR policies OR regulation OR regulations OR legislation OR law OR laws OR statute OR statutes OR institutionalization OR governance)

AND

TS=(pricing OR reimbursement OR funding OR financing OR spending OR “horizon scanning” OR “horizon scan” OR “early awareness” OR “alert system”)

For **Scopus**:

TITLE-ABS-KEY(“health technology assessment” OR HTA OR “value assessment” OR “comparative assessment" OR appraisal OR appraisals)

AND

TITLE-ABS-KEY(“medical device” OR “medical devices” OR “medical technology” OR “medical technologies” OR medtech OR equipment OR procedure OR procedures)

AND

TITLE-ABS-KEY(policy OR policies OR regulation OR regulations OR legislation OR law OR laws OR statute OR statutes OR institutionalization OR governance)

AND

TITLE-ABS-KEY(pricing OR reimbursement OR funding OR financing OR spending OR “horizon scanning” OR “horizon scan” OR “early awareness” OR “alert system”)

1. **Clarifications on the elements of the *framework of the institutionalization of Health Technology Assessment of medical devices in a country***
2. Impact of HTA-MDs recommendations with regard to funding decisions

**Clarification:** This element distinguishes between systems where HTA-MDs findings directly determine funding decisions (binding; regulatory role) versus systems where assessments serve as recommendations that decision-makers may consider but aren't obliged to follow (non-binding; advisory role).

1. Basis for national HTA-MDs institutionalization

**Clarification:** This refers to the foundational authority establishing HTA-MDs activities. Technical procedural documents provide methodological guidance but carry less authority than legal acts, which formally embed HTA in legislation and potentially mandate adherence to assessment outcomes.

1. The body entrusted with the public HTA-MDs mandate can commission external experts for assessment and/or appraisal activities

**Clarification:** This element examines whether the HTA-MDs body can engage external expertise or must rely exclusively on in-house resources. The ability to commission external experts may increase assessment capacity and may bring specialized knowledge for complex technologies.

1. Nature of the body entrusted with the national mandate for HTA-MDs activities

**Clarification:** This describes the nature of the organization conducting HTA-MDs, reflecting different governance approaches.

1. Type of healthcare system

**Clarification:** The underlying healthcare financing model shapes HTA implementation. Beveridge systems (tax-funded, national health services) contrast with Bismarck systems (social insurance-based) and National Health Insurance models, each creating different incentive structures and decision pathways for technology adoption.

1. Prevailing level of HTA-MDs activities

**Clarification:** This indicates whether assessments occur primarily at national, regional, or local levels. National approaches may offer stronger standardization and efficiency, while sub-national approaches may better address local priorities and implementation contexts.

1. Funds allocated specifically for HTA-MDs system implementation (technical/organizational structures and/or HTA-MDs activities)

**Clarification:** This element identifies whether dedicated funding exists for HTA-MDs activities. Specifically allocated funds typically enhance system stability, quality, and independence compared to scenarios where HTA-MDs activities must compete for resources within broader budgets (for HTA of pharmaceuticals and/or other activities).

1. HTAs are mandatory before the decision whether MDs, or the procedures/systems/programs in which they are employed, are eligible for reimbursement/funding

**Clarification:** This distinguishes systems requiring formal assessment before funding decisions from those allowing reimbursement without structured evaluation. Mandatory assessment likely indicates stronger commitment to evidence-informed decision-making.

1. The HTA-MDs body is responsible for the decision on pricing and/or reimbursement tariffs

**Clarification:** This element identifies whether the HTA body itself determines pricing and/or reimbursement, or merely provides information for others to decide. Direct decision authority represents stronger integration of assessment and funding mechanisms.

1. Consultations of external stakeholders to guide design of methodologies

**Clarification:** This examines whether methodology development involves input from stakeholders outside the HTA body. External consultation typically enhances relevance, acceptability, and comprehensiveness of assessment approaches.

1. Kinds of domains of MDs analyzed

**Clarification:** This distinguishes between evaluations examining only clinical aspects (efficacy, safety, health problem and current use of technology, technical characteristics), only non-clinical aspects (economic, organizational, ethical, legal, social), or both.

1. Publicly available assessment and/or appraisal reports

**Clarification:** This element concerns transparency in the HTA process. Public availability of reports enhances accountability, enables scrutiny of methods and conclusions, and supports knowledge dissemination across healthcare systems.

1. Education activities to HTA-MDs stakeholders provided by national HTA body

**Clarification:** This identifies whether the HTA-MDs organization actively builds stakeholder capacity through educational initiatives.

1. Conduction of a systematic process of priority setting of HTA-MDs evaluations (existence of an ordered list; systematic topic identification, selection, prioritization)

**Clarification:** This examines whether formal processes exist for identifying which technologies to assess first.

1. Conduction of a systematic process of Horizon Scanning for MDs

**Clarification:** This refers to structured processes for early identification of emerging technologies, enabling proactive assessment planning and facilitating timely evaluation of promising innovations.

1. Designated appeal mechanisms are in place against HTA-MDs outcomes (placed during/after the assessment process and/or the deliberative process)

**Clarification:** This identifies whether formal processes exist for challenging assessment findings or decisions. Appeal mechanisms enhance procedural fairness, accountability, and opportunities to address potential methodological or interpretive issues.

1. ***Institutionalization of Health Technology Assessment of medical devices in a country for each Cluster***

Here we report the institutionalization of Health Technology Assessment of medical devices in Swiss Confederation (Cluster 1), The Netherlands (Cluster 2), and Denmark (Cluster 3) using the descriptive framework developed for the present study. Data was collected until August 2024.

| **N.** | **Element of HTA-MDs institutionalization** | **Element modality for Swiss Confederation** | **Element modality for The Netherlands** | **Element modality for Denmark** |
| --- | --- | --- | --- | --- |
| i | Impact of HTA-MDs recommendations with regard to funding decisions | Binding results of HTA-MDs | Non-binding results of HTA-MDs | Non-binding results of HTA-MDs |
| ii | Basis for national HTA-MDs institutionalization | Legal acts formalizing procedural documents | Legal acts formalizing procedural documents | Technical procedural documents |
| iii | The body entrusted with the public HTA-MDs mandate can commission external experts for assessment and/or appraisal activities | The body can commission external experts for assessments and/or appraisals, and it is able to run these activities in-house as well | The body can commission external experts for assessments and/or appraisals, and it is able to run these activities in-house as well | The body cannot commission external experts, rather, it relies solely on in-house production of assessments and/or appraisals |
| iv | Nature of the body entrusted with the national mandate for HTA-MDs activities | Governmental | Governmental | Governmental |
| v | Type of healthcare system | Bismarck | Bismarck | Beveridge |
| vi | Prevailing level of HTA-MDs activities | National | National | Sub-national |
| vii | Funds allocated specifically for HTA-MDs system implementation (technical/organizational structures and/or HTA-MDs activities) | The system is financed through funds specifically allocated for HTA-MDs | The system is financed through funds specifically allocated for HTA-MDs | The system is financed through funds specifically allocated for HTA-MDs |
| viii | HTAs are mandatory before the decision whether MDs, or the procedures/systems/programs in which they are employed, are eligible for reimbursement/funding | A HTA evaluation is necessary for determining reimbursability or allocating funds | Even without an available HTA evaluation, reimbursability can be determined and funds can be allocated | Even without an available HTA evaluation, reimbursability can be determined and funds can be allocated |
| ix | The HTA-MDs body is responsible for the decision on pricing and/or reimbursement tariffs | Reimbursement | Reimbursement | None |
| x | Consultations of external stakeholders to guide design of methodologies | Methodologies are designed without the consultation of stakeholders external to the HTA-MDs body | Stakeholders (e.g., clinicians, healthcare experts, patients, developers) external to the HTA-MDs body are consulted to guide the design of methodologies of evaluations | Stakeholders (e.g., clinicians, healthcare experts, patients, developers) external to the HTA-MDs body are consulted to guide the design of methodologies of evaluations |
| xi | Kinds of domains of MDs analyzed | Clinical and non-clinical aspects | Clinical and non-clinical aspects | Clinical and non-clinical aspects |
| xii | Publicly available assessment and/or appraisal reports | Only assessment reports become publicly available | Both assessment and appraisal reports become publicly available | Only assessment reports become publicly available |
| xiii | Education activities to HTA-MDs stakeholders provided by national HTA body | The HTA-MDs body does not organize educational activities on HTA-MDs aimed at stakeholders involved in HTA | The HTA-MDs body does not organize educational activities on HTA-MDs aimed at stakeholders involved in HTA | The HTA-MDs body organizes educational activities on HTA-MDs aimed at stakeholders involved in HTA |
| xiv | Conduction of a systematic process of priority setting of HTA-MDs evaluations (existence of an ordered list; systematic topic identification, selection, prioritization) | The order of initiation of HTA-MDs evaluations is not systematically determined | HTA-MDs evaluations are initiated following an order resulting by processes of topic identification, selection, and prioritization | HTA-MDs evaluations are initiated following an order resulting by processes of topic identification, selection, and prioritization |
| xv | Conduction of a systematic process of Horizon Scanning for MDs | Horizon Scanning activities for MDs are systematically conducted | Horizon Scanning activities for MDs are systematically conducted | Horizon Scanning activities for MDs are not systematically conducted |
| xvi | Designated appeal mechanisms are in place against HTA-MDs outcomes (placed during/after the assessment process and/or the deliberative process) | There are no designated mechanisms through which stakeholders may formally appeal the contents of HTA-MDs reports or decisions concerning evaluated technologies | Designated mechanisms are in place to allow stakeholders to appeal the contents of HTA-MDs reports or decisions concerning evaluated technologies | There are no designated mechanisms through which stakeholders may formally appeal the contents of HTA-MDs reports or decisions concerning evaluated technologies |
